# Designing a methodological system for the development and support of gifted and motivated students

**DOI:** 10.3389/fpsyg.2023.1098989

**Published:** 2023-04-03

**Authors:** Aliya Mambetalina, Timur Nurkeshov, Arstan Satanov, Aigul Karkulova, Erkulan Nurtazanov

**Affiliations:** ^1^Department of Psychology, L. N. Gumilyov Eurasian National University, Nur-Sultan, Kazakhstan; ^2^Principle, Republican Scientific and Practical Center "Daryn" of the Ministry of Education and Science of the Republic of Kazakhstan, Nur-Sultan, Kazakhstan; ^3^Vice Principle, Republican Scientific and Practical Center "Daryn" of the Ministry of Education and Science of the Republic of Kazakhstan, Nur-Sultan, Kazakhstan; ^4^Laboratory of Pedagogical Excellence, Republican Scientific and Practical Center "Daryn" of the Ministry of Education and Science of the Republic of Kazakhstan, Nur-Sultan, Kazakhstan; ^5^Media Support and Publishing Department, Republican Scientific and Practical Center “Daryn” of the Ministry of Education and Science of the Republic of Kazakhstan, Nur-Sultan, Kazakhstan

**Keywords:** comprehensive teaching methods, development of intelligence, gifted children, improving motivation to learn, interactive learning

## Abstract

The paper was intended to develop a new methodological system and test its impact on the development of motivation and giftedness among children. The experiment was conducted among 1,200 children from grades 3, 7, and 10 by researchers from the Daryn Republican Applied Research Center of the Ministry of Education and Science of the Republic of Kazakhstan and L.N. Gumilyov Eurasian National University. The teaching methodology involved: interactive technologies; developing projects with faculty members; and conducting electives in the exact sciences, humanities, natural sciences, and the creative arts. The experiment lasted 4 months. Before and after the experiment, all respondents were evaluated by their instructors according to the following four criteria: academic giftedness, creative giftedness, social giftedness, and intellectual giftedness. The overall result demonstrated an increase in the level of giftedness to above-average values. Motivation levels observed among respondents in grades 3, 7, and 10 were 1.71; 1.72, and 1.54, respectively. The level for this criterion also reached above-average values. This implies that this technique is effective. This technique can already be applied not only in special schools for gifted children, but also in general educational institutions to achieve better results.

## 1. Introduction

Giftedness is a high creative potential, a single and holistic characteristic of a child as a result of systemic interaction of cognitive, motivational, emotional, volitional and other personal characteristics and socio-cultural environment, constituting a particularly favorable internal prerequisite for further development (Mcclain and Pfeiffer, 2012). Gifted children are of great interest because of their great potential and range of abilities. However, they also face challenges which may hinder the development of their talents and abilities (Berg and McDonald, 2018). The development and socialization of gifted and motivated children becomes an important challenge. Children with excellent intelligence may have problems with motivation in learning and socialization among their peers (Kornmann et al., 2015). This is usually because academic programs carry a low cognitive load for gifted children (Tavani et al., 2009). Professionals have expressed concern about the psychological and physical condition of some children with superior intelligence (Cook et al., 2020). In some cases, gifted children suffer from various mental and immune system disorders due to very irregular functioning of the cerebral cortex and some parts of the brain. For example, some gifted children have more activity in the dorsal prefrontal cortex, which is responsible for cognitive functions. At the same time, the ventral prefrontal cortex may be less active. This can cause problems on the psycho-emotional spectrum in children. This can lead to depression, bipolar disorder and attention deficit hyperactivity disorder (Adelodun, 2011). As a result of these factors, children are unable to interact with their peers. Socialization problems often arise because gifted children are simply not interested in interacting with their peers (Pandya, 2021). Gifted children also face the problem of the education system in mainstream schools. Since the standard curriculum does not provide an opportunity to develop the prodigy’s abilities, many lose motivation to gain profound knowledge in various sciences (Dai, 2020). Motivation is one of the most important qualities that contribute to achievement. Developing this factor in children in an educational setting requires increasing enthusiasm for learning (Mayo, 2019). When it comes to wunderkind children, their motivation to learn needs to be developed and supported in order to increase their self-esteem. This relationship is due to the fact that children with strong self-esteem are keen to achieve more because of their openness to information and new ideas. This factor will lead such children to achieve ambitious goals (Berestova et al., 2022). Many different educational strategies for gifted children are being developed. Online learning using interactive technologies and other digital tools is being developed. Many different learning applications and programs for computers, tablets and other devices are also being developed (Palvia et al., 2018). To support children with increased intelligence, many schools are developing electives in different subjects. These can be language, mathematics, literature, creative discussion groups, which usually meet after compulsory school classes (Ting and Lee, 2012). There are also special classes and schools for gifted children where the cognitive load is higher compared to regular schools. These schools quite often focus on a particular area of learning, such as the humanities, mathematics or science (Lee et al., 2021). Gifted children may be more motivated than their peers. This is because children with higher levels of intelligence learn better and perform better. As a consequence, children have an interest in learning new things and in exploring a topic in more depth. This fact should be taken into account when updating the developmental program for children with increased intelligence. Intrinsic motivation correlates with the success of gifted children (Mammadov et al., 2021). Motivation to learn is driven by many factors. The crucial ones include not only the academic program and the cognitive load, but also the relationship of students with instructors, peers and parents (Heilat and Seifert, 2019). Gifted children are not always able to establish relationships with normal peers. Often this is due to the difference of interests. This is another reason to create separate classes and discussion groups for children with superior intelligence. In a team where children with high IQs are taught, it is easier to establish emotionally positive relationships between students (Hennessey, 2005). Family relationships also affect the learning, motivation, and future success of gifted children (Goolsby et al., 2019). Because children with superior intelligence have differences in emotional behavior, a psychologist’s advice might be required in some cases (Cross et al., 2020; Mambetalina et al., 2022). The motivation of children with superior intelligence is also positively affected by their creative thinking. Such a program should include not only learning content, but also various creative projects (making abstracts of presentations on topics of interest to children) in which gifted children can be involved (Güçyeter et al., 2017). However, gifted children have a better chance of succeeding than their peers with a standard level of intelligence. This provides them with better prospects for the future (Abdulla Alabbasi et al., 2021). If the right talent is developed, such children are not only capable of succeeding in their careers but also of creating their own projects. With out-of-the-box thinking, such children can even make discoveries in various fields of science (Ogurlu and Özbey, 2022).

The above suggests that the support and development of gifted children is a fairly important and relevant topic for research and the creation of new techniques.

### 1.1. Literature review

To understand the concepts of learning and their impact on gifted children, the work of other researchers was analyzed.

Researchers from Indiana University and Boston College studied the relationship between effort and outcome, and the impact of these factors on student motivation. The researchers found that low-intelligence and intermediate-intelligence children have to work harder than their gifted peers to achieve their goals. Gifted children have an easier time learning new skills. As a consequence, many gifted children are more motivated, as acquiring knowledge is not a problem for them and they tend to want to go deeper into the topic and expand their knowledge in the context of the topic of interest. Because of their high intelligence they have a greater resource for learning (Muenks and Miele, 2017).

Researchers at Vanderbilt University looked at children with superior intelligence on a case-by-case basis. These researchers have clarified that, despite the emotional lability, child prodigies are able to achieve success in various fields. In their opinion, the instructors’ primary task is to identify capable children and make sure such children develop in a particular area that they are better at (humanities, mathematics, etc.). This will give the child the opportunity to express own talent in a particular area, as many gifted children do not have multi-potentiality (Lubinski, 2016).

Researchers at the University of North Texas (United States) studied the impact of the learning environment on students attending a special school for gifted children. To do this, they conducted a meta-analysis of works on the relevant topic. Particular aspects of the approaches that were considered included the development of children’s freedom of creative thinking. Some sources talked about supplementing the school curriculum. Children engaged in various research projects designed by the pupils themselves, as well as making adjustments to existing good practice. This gave the children an opportunity not only to gain the necessary knowledge but also to learn how to apply it. In conclusion, the analysts concluded that these techniques in combination had a positive effect on the motivation of the pupils (Lee et al., 2021).

Researchers from Belgium and the Netherlands focused on the main paradigms of teaching children with superior intelligence. They were able to identify five crucial components of successful learning for children with superior intelligence: (1) an inclusive approach to learning; (2) a response to educational needs; (3) social constructivism; (4) evidence-based learning; (5) new perspectives on giftedness. Researchers also argued that such a system requires competent professionals to teach gifted children (van Gerven, 2021).

Researchers from the University of the Pacific and the University of California (U.S.) looked at curriculum changes to improve motivation within a group of students. Initially, researchers encountered the phenomenon of poor motivation among students because the program included a superficial study of the course content. Then the teaching methods were changed. In addition to studying various content, student had to find its application in real-world contexts (for example, in his or her future career). By the end of the experiment, the motivation to learn in the studied groups improved significantly (Ditta et al., 2020).

Researchers from Australia and Vietnam also looked at student motivation. They found that poor student motivation was attributed to the lack of instructor feedback. Researchers concluded that instructor feedbacks and a personalized approach to each student significantly improve students’ motivation and learning. This is because the instructor feedback enables students to develop their research skills through various projects on topics of interest to them. The instructor’s openness to the students gives them the opportunity to discuss relevant issues during the course (Maag et al., 2022).

The educational needs of gifted children were also studies in the Junior College of Msida at University of Malta. This study suggested that instructors pay little attention to children with superior intelligence being convinced that such children can handle the tasks themselves. Researchers believe that great achievements require motivation and creative learning in addition to skills (Marks, 2001).

Effects of various educational methods applied to gifted children were studied in the Chinese Academy of Sciences (Beijing). This research compared the effectiveness of the traditional teaching methods and teaching methods relying on multimedia tools. The participants of the experiment were divided into two groups. One group of gifted children was educated according to the standard school curriculum, and the second group of students was educated according to a special curriculum with expanded opportunities and in-depth study of relevant subjects. The research findings suggested that children who attended a special training program began to demonstrate better performance in attention (concentration, distribution, control, stability, and switching) than the group attending the traditional school program (Tao and Shi, 2018).

The interaction between superior intelligence and students’ creativity was studied in Istanbul University (Turkey). The researchers conducted a comparative test analysis of creativity factors between gifted children from special schools and regular students. The findings suggested that fluency, flexibility, creativity and originality are stronger among children with superior intelligence compared to their peers. Based on the findings, the researchers concluded that creativity needs to be developed as well, aside from cognitive skills (Kahveci and Akgul, 2019).

Thomas Bath University (Czech Republic) looked at the phenomenon of giftedness and its impact on socialization. According to researchers, socialization difficulties are no more common among gifted children than among ordinary children. Indeed, gifted students are more likely to be singled out from their peers, but their giftedness may rather be an added advantage. Thus, gifted children can help their peers to learn, which can be an additional social lever, according to Czech scholars (Klimecká, 2022).

Researchers from Valdosta State University (Georgia, United States) looked at the socio-psychological profile among gifted students. The analysis revealed four profiles: resistant, average, overly controlling and calm. The most common profile among gifted children was the stable profile. This indicates a normal level of socialization and emotional well-being. From this the researchers concluded that most gifted children have no problems with socialization (Mammadov, 2022).

The above points out not just to the importance of supporting gifted children, but also to the changes in the education system for them. In this case, the learning system’s primary task involves personalized approach to each student and developing the students’ feedback. Another important aspect includes development of children’s creative thinking and improving their motivation.

### 1.2. Problem statement

This experiment was motivated by the need to develop an educational methodology and to conduct additional research into the development and support of gifted and motivated students, and to evaluate the effectiveness of the chosen approach. The research develops a methodology for teaching gifted children and focuses on the impact of such methodology on children’s development and motivation. The primary task was to introduce the special instruction methodology in special schools. The research findings were evaluated by the instructors of these schools based on student achievements. Creative giftedness, social skills, and intelligence were also taken into account.

## 2. Methods and resources

### 2.1. Research design and sample

The simple random sampling method was used to assess the impact of educational techniques on support and motivation of gifted children. The experiment involved 1,200 children (612 boys and 588 girls) in grades 3, 7, and 10 of special schools. Participants were aged 8 to 17 years. The study was conducted by researchers from the Daryn Republican Applied Research Center, the Ministry of Education and Science of the Republic of Kazakhstan, and L.N. Gumilyov Eurasian National University as part of *Science behind Improvements in Education and Research System* Project. The experiment was implemented in 25 schools that sent applications to participate in the experiment.

### 2.2. Experiment

Before the study began, all participants’ skills were assessed by their instructors. Four main criteria were used in the evaluation: academic giftedness, creative giftedness, social giftedness and intellectual giftedness. Each criterion has its own value, which is composed of a number of factors. For example, academic achievements imply a child’s activity during classes and understanding of the course content. Creative giftedness demonstrates student’s openness to new knowledge, ingenuity, and ability to engage in creative activities (music, art). Social giftedness suggests presence of oratory skills and the ability to find common ground with peers. Intellectual giftedness points to children’s ability to think logically, memorize content, as well as children’s curiosity. J. Guilford’s test, Stanford test, and Mekhrabian test were used to examine the level of giftedness (Ming et al., 2016; Kendrick and Fullerton, 2021; Rossiter, 2022). The J. Guilford test consisted of 14 items. Each of them was presented with a situational picture. There were also three pictures next to each other which implied options to continue the situation. The task of the respondents was to choose what they thought would be the most appropriate way forward. The Stanford test was introduced to assess intelligence and consisted of 20 questions which demonstrated logical reasoning tasks. The Mehrabian test assessed the level of motivation and consisted of 32 questions. They were conducted online in school using tablets that respondents were given for the duration of the test. This test format was to simplify the calculation of results. An instructor-psychologist was present in the classroom during the test. Each criterion was evaluated on a 10-point scale, where 0–4 mean weak manifestation of qualities, 5–7 constitute average result and 8–10 point to superior giftedness. At the end of the experiment, instructors also conducted an evaluation study based on these criteria. The study lasted 4 months. The curriculum in the special classes has been improved, while the timetable has remained the same. The changes concerned the teaching methodology and the assignments given to the pupils. Classes were conducted in accordance with the regular school curriculum. The methodology covered all subjects of the school curriculum (sciences, humanities and natural sciences) equally. All lessons were interactive, with many presentations and videos (Bourbour, 2023). The main points of the program will be outlined point by point.

1. Teachers prepared learning materials and displayed them on the interactive whiteboard using an overhead projector. Also, for each lesson, one of the children prepared a presentation to reveal the importance of the topic and to provide different interesting facts (Shi et al., 2021).
2. At the end of each week, students had a homeroom meeting where children, having chosen a topic of interest to them, prepared reports using different sources. While preparing during the week, the children had to consult with the teachers after class about their work. As one homeroom meeting could not accommodate all of the class students’ project presentations, it was decided to present five pieces of work per week. Accordingly, during the week 5 pupils from the class prepared the presentations. The order of the projects was chosen according to a list or by choice in each class, but each student had to make a presentation during the experiment. Teachers had to prepare the projects together with the students during these presentations. Students involved in the projects had to meet in class with the teacher and discuss project ideas after class for 1 week. The teacher advised the students on how to complete the work, what literature they needed, and students could ask questions (Ozcan, 2016).
3. During the experiment, each school held electives in different areas (science, science, languages and creative discussion groups; Vreys et al., 2018). They were conducted by guest lecturers from universities in the city. Participation in the electives was compulsory, but the direction the students chose themselves. These classes were held twice a week for two classroom hours after school. The science electives included a review of mathematics, physics and chemistry in years 7 and 10. Students in Grade 3 had only a maths elective. The 2 h of the elective were divided with a break of 15 min. The first hour dealt with theory and application of science. Problem solving and discussion of emerging issues took place during the second class hour. Humanities electives included the study of foreign languages (English, German, French). These extracurricular activities also included an introduction to the culture of the regions. The first hour in these elective classes included vocabulary and grammar, and the second hour included cultural introduction and conversation practice. The natural sciences were represented by biology and natural history. Creative electives included dance classes with a choreographer and additional visual arts classes. All respondents were in the same groups as before the experiment. However, the groups in the elective classes were formed randomly. In this case they were divided exclusively according to age category. The duration of the experiment was 4 months. The attendance rate was to be 90% or more.

### 2.3. Statistical processing and data analysis

A special statistical analysis program SPSS 26.0 was used to process the results of the experiment. The results were interpreted and visualized in Microsoft Excel 2019. Student’s *t*-test helped to compare the effectiveness of the proposed training in groups and the impact of the technique on the overall outcome of the study. Using Student’s *t*-test, the level of mean pre-test and final test score in all control groups was assessed for the factors sought. These included 5 main items: academic giftedness, creative giftedness, social giftedness, intellectual giftedness and motivation. The scores after the test were compared and the study revealed a difference between the groups. The test was found to be statistically significant (*p* ≤ 0.05). 95% confidence intervals (CI) were calculated for median analysis.

### 2.4. Limitations

The study is based on simple random sampling, where not all participants had superior giftedness. All findings are presented as the average of the entire sample. Therefore, it is impossible to understand exactly how this method will affect the development of a particular individual with particular cognitive skills. The methodology also includes attending electives, but this is completely voluntary. Therefore, it is not possible to fully assess the impact of this part of the methodology. First, there are many different areas that affect children’s development to varying degrees. Second, extracurricular activities are voluntary, and not all participants may be interested. All of these factors should be taken into account when considering the results of this study.

### 2.5. Ethical issues

This study required the respondents’ consent. Since all participants are under 18 years of age, permission to participate was requested not only from them, but also from their parents and guardians. All parents were informed verbally and in writing about the conduct and details of the experiment. Parents of participants signed the relevant documents, consenting to their child’s participation in the study. Respondents were also informed about the experiment and its conditions. The children themselves also signed a consent to participate in the study.

## 3. Results

Prior to the experiment, instructors at the schools where the research took place evaluated their students based on academic giftedness, creative giftedness, social giftedness, and intellectual giftedness criteria. All results are presented as a group average. For 3rd graders, the academic giftedness criterion was 6.52 out of possible 10 points. The 3rd graders’ creative giftedness score was 6.44. Social giftedness score was 7.47. 3rd graders’ intellectual giftedness scored 6.72 out of 10 points. 3rd graders’ motivation was 6.54 points.

7th graders scored 6.97 out of 10 points on the academic giftedness and 6.82 points in on the creative giftedness. The social giftedness coefficient was 7.23 out of possible 10 points. The intellectual giftedness score was 6.89 points. Motivation among 7th graders was 6.45 out of possible 10 points.

10th graders scored 7.12 points in academic giftedness and 7.05 points in creative giftedness. Social giftedness score among 10th graders was 7.52 points. Intellectual giftedness score was 7.08 points at the preliminary phase, and motivation score—6.88 points. This suggests that all groups demonstrated the average level of giftedness and motivation in all criteria. Table 1 summarizes all preliminary findings.

**Table 1:** Preliminary findings on student giftedness.

Assessment criteria	Grade 3	Grade 7	Grade 10
Academic giftedness	6.52 points	6.97 points	7.12 points
Creative giftedness	6.44 points	6.82 points	7.05 points
Social giftedness	7.47 points	7.23 points	7.52 points
Intellectual giftedness	6.72 points	6.89 points	7.08 points
Motivation	6.54 points	6.61 points	6.88 points

At the end of the experiment, instructors conducted a final assessment of the students’ giftedness. 3rd graders scored 8.07 out of possible 10 points in academic giftedness. The creative giftedness score was 8.03 points, which is the average for the group. Social giftedness score increased to 8.85 out of 10 points. Intellectual giftedness among 3rd graders increased to 8.23 out of possible 10 points. Motivation score was 8.25 points. Finally, by the end of the experiment 3rd graders had above-average scores in giftedness.

7th graders had academic giftedness score of 8.27 out of 10 points. Creative giftedness increased to 8.32 points. By the end of the study, social giftedness score reached 8.47 points. Intellectual giftedness rose to 8.35 out of possible 10 points. Motivation improved to 8.33 points. Scores earned by 7th graders in giftedness also increased to an above-average level. This suggests a positive trend within this methodology.

10th graders improved their academic giftedness score to 8.89 out of possible 10 points. Creative giftedness increased to 8.09 points. By the end of the study, the social giftedness score was 9.04 points. Intellectual giftedness among 10th graders rose to 8.38 points. Motivation improved to 8.42 points. By the end of the study, 10th graders’ giftedness in all criteria was above average. These findings were obtained by calculating the entire sample’s mean. Table 2 provides the findings of the final study.

**Table 2:** Preliminary findings on student giftedness.

Assessment criteria	Grade 3	Grade 7	Grade 10
Academic giftedness	8.07 points	8.27 points	8.89 points
Creative giftedness	8.03 points	8.32 points	8.09 points
Social giftedness	8.85 points	8.47 points	9.04 points
Intellectual giftedness	8.23 points	8.35 points	8.38 points
Motivation	8.25 points	8.33 points	8.42 points

After the final test, the results were tested using Student’s *t*-test. The “before” and “after” progress was compared for each class separately. The significance level of all criteria was below 0.05 and in some cases below 0.01. The null hypothesis was rejected. This demonstrates a significant level of variation over the course of the study. Significant changes were found in the motivation indicator among 10th grade students. Creativity increased significantly in grades 7 and 3. Also among the respondents of class 3, in addition to creativity, the criteria of intellectual and akdemic giftedness increased significantly. This suggests plasticity and responsiveness in the different age groups. The results are shown in Tables 3–5.

**Table 3:** Calculating class 3 results using student’s *t*-test.

Assessment criteria	Grade 3 before	Grade 3 after	Significance level (*p*-value)
Academic giftedness	6.52 ± 0.141	8.07 ± 0.141	0.008**
Creative giftedness	6.44 ± 0.141	8.03 ± 0.141	0.008**
Social giftedness	7.47 ± 0.141	8.85 ± 0.071	0.017*
Intellectual giftedness	6.72 ± 0.141	8.23 ± 0.141	0.009**
Motivation	6.54 ± 0.141	8.25 ± 0.141	0.007*

* *p* < 0.05.

** *p* < 0.01.

**Table 4:** Calculating class 7 results using student’s *t*-test.

Assessment criteria	Grade 7 before	Grade 7 after	Significance level (*P*-value)
Academic giftedness	6.97 ± 0.141	8.27 ± 0.141	0.012*
Creative giftedness	6.82 ± 0.141	8.32 ± 0.141	0.009**
Social giftedness	7.23 ± 0.141	8.47 ± 0.141	0.012*
Intellectual giftedness	6.89 ± 0.141	8.35 ± 0.071	0.017*
Motivation	6.61 ± 0.141	8.33 ± 0.071	0.013*

* *p* < 0.05.

** *p* < 0.01.

**Table 5:** Calculating class 10 results using student’s *t*-test.

Assessment criteria	Grade 10 before	Grade 10 after	Significance level (*p*-value)
Academic giftedness	7.12 ± 0.071	8.89 ± 0.141	0.013*
Creative giftedness	7.05 ± 0.071	8.09 ± 0.141	0.027*
Social giftedness	7.52 ± 0.141	9.04 ± 0.071	0.016*
Intellectual giftedness	7.08 ± 0.071	8.38 ± 0.141	0.019*
Motivation	6.88 ± 0.141	8.42 ± 0.141	0.009**

* *p* < 0.05.

** *p* < 0.01.

The research findings suggest the effectiveness of this methodology for the experiment. Since all indicators among all grades moved to above-average giftedness, this implies that methodology is suitable for teaching not only gifted children, but also children with average giftedness. Moreover, this technique has a positive effect on cognitive skills among children with average giftedness. Instructors also draw attention to students’ improved motivation to learn. One of the instructors mentioned that with this method children’s interest in learning has increased significantly, and some children even began to think about future careers and personal development options.

However, the study relied on simple random sampling and was conducted in a large focus group. The experiment was conducted not only among gifted children, and all findings are presented as a mean value. Therefore, they do not imply any well-defined results. Additional studies of implementation of this technique may be required.

## 4. Discussion

Researchers from Oslo, Norway, studied the influence of instructor competence and assistance on the students’ emotional component. In this experiment, they looked at the impact of the instructor-student relationship. Findings suggested strong impact of the instructor’s role in the learning process. The researchers concluded that the instructor’s role in the learning environment is one of the most important and quite strongly affects the students’ achievements (Ekornes, 2017). Comparison of this finding with the results of the experiment implies that the interaction between instructors and children during the learning process plays a core role in their academic achievements and motivation to learn. The differences in the research come from the fact that in this study the instructor-student relationship is only part of the developed methodological program.

Researchers from Turkey studied the impact of the relationship between instructors and students on the latter’s academic achievements. The experiment’s main task was to change the approach to the learners. Emphasis was placed on building collaboration between instructors and students. This was expressed in development of various projects with the instructors’ support. The findings suggested that with the instructors’ support and interest in learning, students’ achievements and learning motivation improve (Altan and Sağlamel, 2015). Instructor-student interaction techniques are also discussed in this study. The above implies the effectiveness of this methodology in the education system. The only difference is that in this experiment it is only part of the overall methodology.

Researchers at the University of Northern Colorado (United States) studied the impact of interactive technology on student motivation. They concluded that the introduction of interactive technology (learning applications for PCs and tablets) increases motivation to learn. The increase in motivation has been attributed to the ease of use of the techniques in the learning environment. Interactive technologies are also believed to improve concentration. However, in addition to this, the effectiveness of the technique depends on the interaction of the parties in the learning process. When interactive technology was used, respondents reported greater motivation and enthusiasm for learning. Also, according to scientists, the motivation could be due to the interest in novelty among the learners (Tsai et al., 2021). Drawing an analogy, it can be observed that interactive technology increased interest in learning from the perspective of this study. However, the difference in the research is due to the choice of interactive learning method. The American researchers discussed the impact of online classrooms, whereas this study looked at multimedia tools used in the classroom.

Researchers from Taiwan compared the impact of interactive technology on children’s academic achievements. For this purpose, a quasi-experiment was conducted, where two groups of children studied the same topic, but in different ways. The first group relied on traditional learning methods, while the other group used electronic books. Ultimately, the group which used the interactive technology performed better. Instructors also mentioned an increased motivation among group members (Sung et al., 2022). This makes it clear that incorporating interactive technology into learning improves both the learning itself and motivation. The difference in the experiment comes from the fact that researchers from Taiwan did not attach value to importance to the relationship between children and instructors, and studied only the impact of interactive technology on student performance.

Researchers from Norway studied the impact of math electives on students’ learning. By conducting an experiment, they found that electives broaden students’ knowledge of the subject and help them gain not only new knowledge, but also learn the ways to apply such knowledge. This fact improves achievements and enhances learning motivation (Radmehr et al., 2022). The current study also describes the available electives, providing an opportunity for students’ self-determination. The difference also comes from the fact that the Norwegian case study deals only with full-time science electives, whereas this paper describes electives in a variety of areas.

Most studies on these methods describe positive trends in their effectiveness. This study also examines the positive impact of a combination of interactive teaching methods, teacher-student interaction on students’ skills and motivation. However, the study relied on a simple random sample distribution and had a large sample of participants. Therefore, the data were not well defined. Also, the personalized effect of this method on each student could not be determined. More research is needed to make a more accurate assessment. Nevertheless, the methodology can be used in special schools for teaching and motivating gifted children. The methodology can also be used to improve learning in ordinary schools.

## 5. Conclusion

The experiment findings pointed to increased academic giftedness among students in grades 3, 7, and 10 by 1.55, 1.30, and 1.77, respectively. Ultimately, overall academic giftedness scores rose to above-average levels. The creative giftedness scores among respondents in grades 3, 7, and 10 increased by 1.59, 1.50, and 1.04, respectively. The overall scores also had above-average values. Social giftedness among students in grades 3, 7, and 10 changed by 1.38, 1.24, and 1.52 points, respectively. The findings for this criterion also became equivalent to an above-average giftedness score. The intellectual giftedness indicator among students in grades 3, 7, and 10 increased by 1.51, 1.46 and 1.30, respectively. The overall result demonstrated an increase in the level of giftedness to above-average values. Motivation levels observed among respondents in grades 3, 7, and 10 were 1.71, 1.72, and 1.54, respectively. The level for this criterion also reached above-average values. The experiment suggested the effectiveness of this method. However, the study relied on simple random sampling with a large sample of participants. Furthermore, not all participants demonstrated strong giftedness before the experiment began. Therefore, this fact does not imply the absolute accuracy of the findings. The research findings are important for further studies addressing development and application of a comprehensive methodology in the learning environment to improve motivation and support for gifted children. Since the study is not without gaps, this fact gives grounds for new research, more specifically by applying this teaching method to smaller focus groups or on a personalized basis. This methodology may also be upgraded, but this would also require additional research in this area. The practical implications of this study involve the possibility to apply this methodology not only in special schools for gifted children, but in regular schools as well.

## Data availability statement

The original contributions presented in the study are included in the article/supplementary material, further inquiries can be directed to the corresponding author.

## Ethics statement

The study was conducted in accordance with the ethical principles approved by the Human Experiments Ethics Committee of L. N. Gumilyov Eurasian National University (Protocol No 6 of 12.02.2021). Since all participants are under 18 years of age, permission to participate was requested not only from them, but also from their parents and guardians.

## Author contributions

AM, TN, AS, EN, and AK were performed the material preparation, data collection, and analysis. AM wrote the first draft of the manuscript. All authors contributed to the study conception and design, and read and approved the final manuscript.

## Funding

This study was prepared within the program trust fund study OR 11465474 “Scientific foundations of modernization of the education system and science” (2021–2023).

## Conflict of interest

The authors declare that the research was conducted in the absence of any commercial or financial relationships that could be construed as a potential conflict of interest.

## Publisher’s note

All claims expressed in this article are solely those of the authors and do not necessarily represent those of their affiliated organizations, or those of the publisher, the editors and the reviewers. Any product that may be evaluated in this article, or claim that may be made by its manufacturer, is not guaranteed or endorsed by the publisher.
